# Colostomy

**Published:** 1907-11-02

**Authors:** 


### COLOSTOMY.

(*Continued from p. 88.*)

Twenty years ago the only form of colostomy performed by surgeons was that which we know as "lumbar" colostomy. In recent times it has been almost replaced by inguinal colostomy. The credit of having brought about this change is due almost entirely to the late Mr. Allingham. As early as 1892 he pointed out the disadvantages of the former method. To quote his own words: "There are many reasons for preferring inguinal to lumbar colostomy. The opening is in front, and can be attended to by the patient himself with far greater facility than when it is in the lumbar region. Further, a pad or truss can be readily adjusted to the opening in the groin. The inguinal operation can be performed with greater ease; the patients usually get well much more quickly and there is less risk of opening any other viscus than the colon. In all these points the inguinal is an advance upon the lumbar operation." The recognition of the truth of these remarks has led to the almost complete abandonment of the lumbar operation, and it is so rarely performed nowadays that it need not be described.

In all cases in which the cause of the obstruction is situated in the rectum or in the lower part of the sigmoid, the best operation is left inguinal colostomy. In advising a patient to undergo colostomy it is necessary to explain to him very exactly what is about to be done, and to leave the final decision to him.

A small incision should be made, at right angles to a line drawn from the left anterior superior spine to the umbilicus. Its centre should correspond to the junction of the outer and middle thirds of this line. It should be about two inches in length. It is far better to split the muscles of the abdominal wall in the direction of their fibres than to cut them across as used to be done. A quadrate opening is given by this manœuvre which affords quite sufficient access to the peritoneal cavity and a much firmer scar is obtained. When the parietal peritoneum has been opened a finger is inserted and the colon is sought for. It can usually be found quite easily by sweeping the finger round towards the crest of the ilium, since the sigmoid tends to lie to the outer side of the wound. When found a small portion is brought up into the wound: this should be examined to see if it is colon, and not small intestine, that has been exposed. The sigmoid is recognised by its longitudinal bands, and its appendices epiploicæ, as opposed to the small intestine which is uniformly round and smooth. This may seem a small point to lay stress on, but it is really an important one, since, if the small gut were fixed in the wound and opened, the results would be disastrous. The sigmoid has a long mesentery which allows of its being brought up with comparative ease. The next point is to pull down the upper limb of the loop until it is taut. This prevents the subsequent occurrence of procidentia, which is a very troublesome complication. Any excess of gut thus produced is returned to the pelvis through the lower half of the wound. The mesentery is now pierced at a point where no veins can be seen by a pair of Spencer-Wells forceps, the point being directed from the middle line towards the outer side. The blades of the forceps are opened and a plug of gauze is clipped between them. The object of this is to retain the forceps in position. If it is introduced from the outer side it tends to slip out during convalescence. A stitch or two may be inserted to fix the serous and muscular coats of the exposed intestine to the skin at each end of the wound. In the absence of acute obstruction the wound is covered with green protective, and is not opened until the following day, when the colon in the wound is shut off from the general peritoneal cavity. The intestine can then be opened with scissors in the long axis of the bowel, and any redundant intestinal wall should be cut away. Some bleeding generally takes place, but it is not of a serious nature, and usually stops of its own accord. To obviate this some authorities have suggested opening the bowel transversely, so as to avoid dividing the vessels which encircle it, but this method does not give so good an opening. The patient experiences no pain when the intestine is opened, and no anæsthetic is required. Some intestinal mucus is often discharged with the fæces: this is a normal event, and need give no cause for alarm. If the obstruction has become acute, it may be necessary to open the bowel at the time of the operation. This is best done by means of a Paul's tube, which is sewn into the gut with a purse string suture. A long tube of thin india-rubber is attached to its other end, so that the fæces are kept out of contact with the wound. The tube generally works its way out about the third or fourth day after the operation. Another method recently suggested for obtaining a good spur is to scratch an opening in the mesentery, and to unite the centre of the skin wound behind the intestine with one or two silk-worm gut sutures. This effectually prevents it from receding into the abdomen; but has the disadvantage that unless the wound is rather larger than usual the intestine may be pinched. The patient may begin to get up at the end of the third week. He must, of course, be provided with a colostomy belt. The best apparatus is one which consists of a celluloid cup to fit the opening. This is kept in position by an elastic or india-rubber belt which ensures its being firmly adjusted to the abdominal wall. The patient soon learns to look after it for himself.

(*To be concluded.*)

